# Supplementary material for: Mutation induced infection waves in diseases like COVID-19
Source: Sci Rep. 2022 Jun 10;12:9641. doi: 10.1038/s41598-022-13137-w (PMC9186490; doi:10.1038/s41598-022-13137-w)
Supplement: Supplementary file 1 — Supplementary Information. [file 41598_2022_13137_MOESM1_ESM.pdf]

Supplementary information: Mutation induced infection waves in diseases like COVID-19

Fabian Jan Schwarzendahl,[1] Jens Grauer,[1] Benno Liebchen,[2] and Hartmut Löwen[1]

[1]*Institut für Theoretische Physik II: Weiche Materie, Heinrich-Heine-Universität Düsseldorf, 40225 Düsseldorf, Germany*

[2]*Institute of Condensed Matter Physics, Technische Universität Darmstadt, Darmstadt, Germany*

(Dated: May 9, 2022)

I. MAXMIMUM OF WAVES IN THE CONSTANT MUTATION RATE MODEL

To obtain some analytical an understanding of the scaling of the maxima in the constant mutation model, we start by considering the equations of susceptible and infections which read

$$\dot{S} = -(\beta_0 + \mu t)SI, \tag{S1}$$
$$\dot{I} = (\beta_0 + \mu t)SI - \gamma I, \tag{S2}$$

where the recovered R follow from $S + I + R = 1$. Redefining the time variable $t' = (\beta_0 + \mu t)$ yields

$$\mu\dot{S} = -t'SI, \tag{S3}$$
$$\mu\dot{I} = t'SI - \gamma I, \tag{S4}$$
$$\tag{S5}$$

where the dot now denotes derivatives with respect to t'. Maxima can be found using $\dot{I} = 0$, which is explicity

$$S^* t'^* = \gamma \tag{S6}$$

where S^* are the susceptible at the maximum and t'^* is the time at which the maximum appears. Taking a derivate of S^* with respect to t'^* gives

$$\dot{S}^* = -\frac{\gamma}{(t'^*)^2}, \tag{S7}$$

which is only valid between maxima. Since the susceptible decay monotonically in time, we assume that the S^* also decays monotonically with

$$\dot{S}^* \sim -S^* t'^* I^*, \tag{S8}$$

where I^* are the infected a the maximum. Using Eq. (S7) and Eq (S8) then yields the scaling

$$I^* \sim \frac{\mu}{(\beta + \mu t'^*)^2}. \tag{S9}$$

II. MATHEMATICAL ANALYSIS OF PHASE DIAGRAM IN BEYOND CONSTANT MUTATION RATE APPROACH

The dynamical equations of our model which goes beyond a constant mutation rate are given by

$$\dot{S} = -\beta SI, \tag{S10}$$
$$\dot{I} = \beta SI - \gamma I, \tag{S11}$$
$$\dot{\beta} = \lambda I, \tag{S12}$$
$$\tag{S13}$$

where R follows from $S+I+R=1$. Dividing Eq. (S10)-(S11) by Eq. (S12) gives

$$\frac{dS}{d\beta} = -\frac{\beta S}{\lambda}, \tag{S14}$$

$$\frac{dI}{d\beta} = \frac{\beta S}{\lambda} - \frac{\gamma}{\lambda}, \tag{S15}$$

where β reparameterises time. Equation (S14) is solved by

$$S = S_0 e^{\frac{\beta_0^2-\beta^2}{2\lambda}}, \tag{S16}$$

with initial susceptible S_0 and initial infection rate β_0. Equation (S15) can be rewritten as

$$dI = \frac{\beta S}{\lambda} d\beta - \frac{\gamma}{\lambda} d\beta \tag{S17}$$

$$= -dS - \frac{\gamma}{\lambda} d\beta, \tag{S18}$$

where we used Eq. (S14). Integrating then yields

$$I - I_0 = S_0 - S - \frac{\gamma}{\lambda}\left(\beta - \beta_0\right) \tag{S19}$$

To obtain the phase diagram shown in the main text, we need to look for the extrema of the infections. Extrema of I are given by the condition $\dot{I} = 0$ which explicitly read

$$S^*\beta^* - \gamma = 0, \tag{S20}$$

where S^* are the susceptible at the extremum and β^* is the infection rate at the extremum. Using Eq. (S16) gives

$$\frac{\gamma}{\beta^*} = S_0 e^{\frac{\beta_0^2-(\beta^*)^2}{2\lambda}}, \tag{S21}$$

which is solved by

$$\beta^* = -i\sqrt{\lambda}\left(W\left(-\frac{\gamma^2}{\lambda S_0^2} e^{-\frac{\beta_0^2}{\lambda}}\right)\right)^{1/2} \tag{S22}$$

where $W(*)$ is the Lambert function. The argument in the Lambert function of Eq (S22) is smaller than zero. This gives rise to two solutions, implying two extrema of the infections. Explicitly the extrema I^* can be found by using

$$I^* = I_0 + S_0 - \frac{\gamma}{\beta^*} - \frac{\gamma}{\lambda}\left(\beta^* - \beta_0\right), \tag{S23}$$

together with Eq (S22). Furtermore, to determine maxima and minima we can use

$$\ddot{I}|_{S^*\beta^*=\gamma} = (I^*)^2 S^*(\lambda - (\beta^*)^2) \tag{S24}$$

such that the sign of $(\lambda - (\beta^*)^2)$ decides between minimum and maximum.

III. CRITICAL SHORT TIME BEHAVIOR IN BEYOND CONSTANT MUTATION RATE APPROACH

At small times we can approximate the number of susceptible people by $S \approx 1$, which gives the set of equations

$$\dot{I} = \beta[I]I - \gamma I, \tag{S25}$$

$$\dot{\beta} = \lambda I, \tag{S26}$$

with the initial condition $\beta(0) = \beta_0$. By changing the initial condition of Eq.(S26) to $\beta(0) = \beta_0 - \gamma$ we can rewrite Eq.(S25) as

$$\frac{d\ln I}{dt} = S_0\beta[I]. \tag{S27}$$

We now use the transformation $\omega = \ln I$, giving

$$\frac{d\omega}{dt} = S_0\beta\left[e^{\omega}\right] \tag{S28}$$

$$\dot{\beta} = \lambda e^{\omega}. \tag{S29}$$

Taking a derivatite of Eq.(S28), gives

$$\frac{d^2\omega}{dt^2} = S_0\dot{\beta} = S_0\lambda e^{\omega}, \tag{S30}$$

which has initial conditions $\omega(0) = \ln(I(0))$ and $\dot{\omega}(0) = \beta - \gamma$ and can be solved explicitly. Putting back the transformation, we arrived at the following expression for the number of infected people

$$I(t) = -\frac{\delta_1}{\lambda\left[1 + \cosh\left(2\ln\delta_2 + t\sqrt{\delta_1}\right)\right]}, \tag{S31}$$

with $\delta_1 = -2I_0\lambda + (\gamma - S_0\beta_0)^2$ and $\delta_2 = -\frac{\sqrt{\gamma - S_0\beta_0 + \sqrt{\delta_1}}}{\sqrt{-\gamma + S_0\beta_0 + \sqrt{\delta_1}}}$. Equation (S31) has poles at

$$t_c = \frac{-2\log(\delta_2) + i\pi(2k-1)}{\sqrt{\delta_1}}, \tag{S32}$$

where k is an integer and the physically relevant critical time t_c is given by k yielding the smallest positive t_c. Expanding Eq. (S31) around the critical time t_c gives

$$I(t) = \frac{2}{\lambda}\frac{1}{(t-t_c)^2}. \tag{S33}$$

IV. INDEPENDENT PARAMETERS OF COARSE GRAINED MSIR MODEL

The equations of our constant mutation rate coarse grained model are

$$\dot{S} = -\beta SI, \tag{S34}$$

$$\dot{I} = \beta SI - \gamma I, \tag{S35}$$

$$\dot{\beta} = \mu. \tag{S36}$$

From the condition $S + I + R = 1$ it then follows that $R(t = 0) = 1 - I_0 - S_0$, such that we have three parameters I_0, S_0 and β_0 for the initial condition. Additionally, we have the parameters μ and γ, making our parameter space five dimensional.

We continue considering the units of each parameter and variable which are $[S] = 1$, $[I] = 1$, $[\beta] = 1/\text{time}$, $[\beta_0] = 1/\text{time}$, $[\gamma] = 1/\text{time}$ and $[\mu] = 1/\text{time}^2$. By nondimensionalizing Eq.(S34)-(S36) we find

$$\dot{\mathcal{S}} = -\beta\mathcal{S}\mathcal{I}, \tag{S37}$$

$$\dot{\mathcal{I}} = \beta\mathcal{S}\mathcal{I} - \mathcal{I}, \tag{S38}$$

$$\dot{\beta} = \mu/\beta_0^2. \tag{S39}$$

with $\mathcal{S} = S\beta_0/\gamma$, and $\mathcal{I} = I\beta_0/\gamma$. The parameter space then reduces to three dimensions where the nondimensional parameters are the initial infected I_0, the basic reproduction number $S_0\beta_0/\gamma$ and the mutation rate μ/β_0^2. The calculation for our model that goes beyond a constant mutation rate is analogous, with the resulting nondimensional mutation rate λ/β_0^2.

V. ESCAPE OF IMMUNITY

A. Steady state

In the steady state the equations including immune escape read

$$0 = -\beta(t,I)SI + \omega R, \tag{S40}$$

$$0 = \beta(t,I)SI - \gamma I, \tag{S41}$$

$$0 = \gamma I - \omega R. \tag{S42}$$

Solving Eqs.(S40)-(S42) together with $S + I + R = 1$, yields

$$S = \frac{\gamma}{\beta}, \tag{S43}$$

$$I = \frac{1 - \frac{\gamma}{\beta}}{1 + \frac{\gamma}{\omega}}, \tag{S44}$$

$$R = \frac{\gamma}{\omega} \frac{1 - \frac{\gamma}{\beta}}{1 + \frac{\gamma}{\omega}}. \tag{S45}$$

$$\tag{S46}$$

In the long time limit β becomes very large for both mutation models, such that γ/β is effectively zero and we find

$$S_\infty = 0, \tag{S47}$$

$$I_\infty = \frac{1}{1 + \frac{\gamma}{\omega}}, \tag{S48}$$

$$R_\infty = \frac{1}{1 + \frac{\omega}{\gamma}}, \tag{S49}$$

$$\tag{S50}$$

which shows a nonzero steady state solution for the infected.

B. Infection waves from linear stability

We start by considering our coarse grained beyond constant mutation rate model with immune escape, which reads

$$\dot{S} = -\beta S I + \omega R, \tag{S51}$$

$$\dot{I} = \beta S I - \gamma I, \tag{S52}$$

$$\dot{R} = \gamma I - \omega R, \tag{S53}$$

$$\dot{\beta} = \lambda I. \tag{S54}$$

We now linearize the system of equations (S51)-(S54) around the state $S = S_0 + \delta S$, $I = I_0 + \delta I$, $R = R_0 + \delta I$ and $\beta = \beta_0 + \delta\beta$, where we neglect term of δ^2. This results in the following system of equations

$$\frac{\mathrm{d}}{\mathrm{d}t} \begin{pmatrix} \delta S \\ \delta I \\ \delta R \\ \delta\beta \end{pmatrix} = \underbrace{\begin{pmatrix} -\beta_0 I_0 & -\beta_0 S_0 & \omega & -S_0 I_0 \\ \beta_0 I_0 & \beta_0 S_0 & 0 & S_0 I_0 \\ 0 & \gamma & \omega & 0 \\ \lambda & 0 & 0 & 0 \end{pmatrix}}_{=\mathcal{M}} \begin{pmatrix} \delta S \\ \delta I \\ \delta R \\ \delta\beta \end{pmatrix}, \tag{S55}$$

where we defined the stability matrix $\mathcal{M}$. In Fig. S1 we show the imaginary part of the four eigenvalues of $\mathcal{M}$ as a function of the rate of immunity escape ω, which are nonzero. Therefore, we expect the system to show oscillations, which are the infection waves shown in Fig. 5(c) (main text). Note that the oscillations are induced by the escape of immunity and are also present with a constant β or a constant mutation rate.

VI. VACCINATION WITH NON-CONSTANT ROLLOUT

Vaccinations are typically not distributed in the public with a constant rate, as assumed in our main text. The vaccination rate is for example influenced by the rate of production and the willingness of the public to get vaccinated. To see what effect a non constant rollout has, we assume the following dynamic infection rate

$$\alpha_{eff} = \frac{\alpha_0}{\sqrt{2\pi}} e^{-(t-t_{max})^2/(2w^2)}, \tag{S56}$$

where we chose the time of maximal vaccination $t_{max} = 5$ weeks, the width of the distribution to be $w = 2$ weeks and α_0 is the bare vaccination rate.

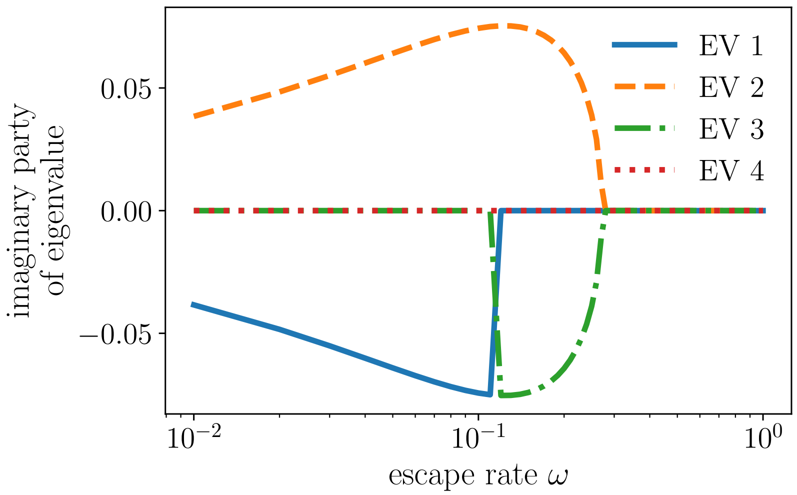

FIG. S1. Imaginary part of the four eigenvalues obtained the stability matrix $\mathcal{M}$. (here $R_0 = 1.2$, $\mu/\beta_0^2 = 2$, $I_0 = 2 \times 10^{-4}$)

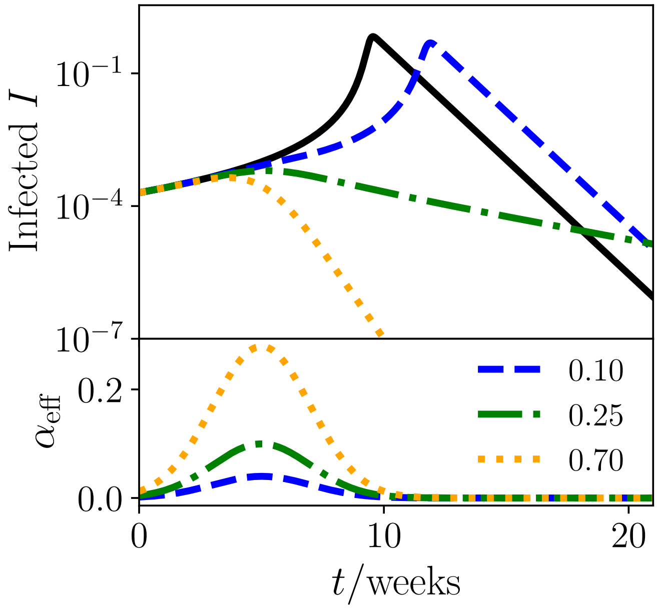

FIG. S2. (Top) Infections as function of time of different α_0. (Black line has no vaccination. $R_0 = 1.2$, $\lambda/\beta_0^2 = 50$, $I_0 = 2{\times}10^{-4}$). (Bottom) Effective infection rate as function of time for different α_0.

In Fig. S2(Top) we show the infected as a function of time with the vaccination rollout specified in Eq.(S56) (see Fig. S2(Bottom)) for our beyond constant mutation rate model. The resulting infected show a behavior that is very similar to the constant vaccination rate model shown in our main text Fig. 5(b). Hence we conclude that the vaccination rollout Eq. (S56) does not induce qualitative changes.

VII. DYNAMICS OF THE INFECTION RATE

A. Constant mutation rate

In our multi component model we constantly draw a new infection rates β_n, which evolve according to the distribution Eq. 7 (main text). In Fig. S3 we show the evolution of the infection rate with time, which shows a linear increase

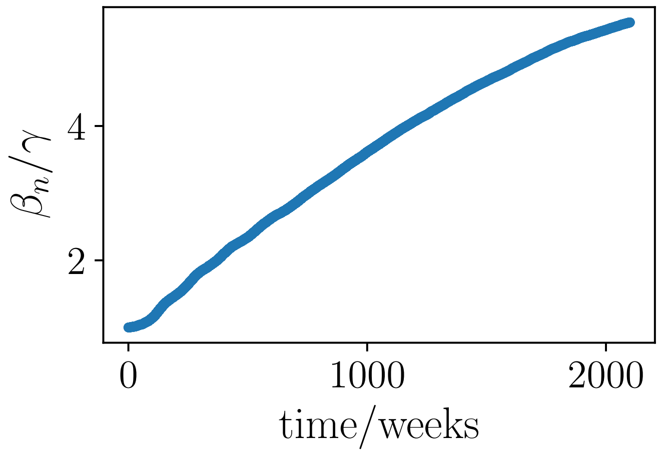

FIG. S3. Infection rates β_n as function of time for our multi component constant mutation rate model.

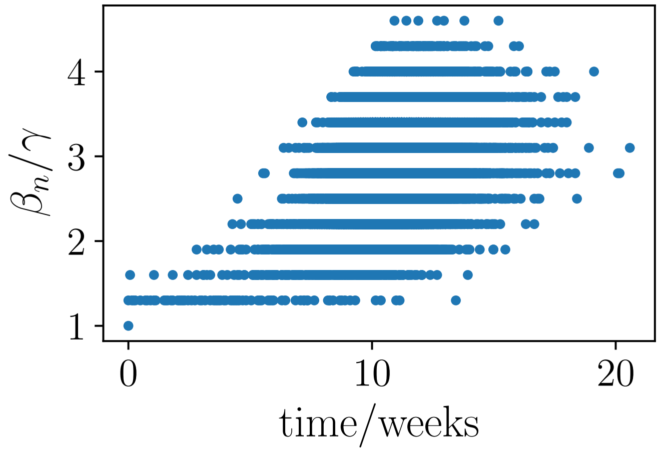

FIG. S4. Infection rates β_n as function of time for our multi component model beyond a constant mutation rate.

of the mean infection rate. This behavior is consistent with the linear increase of our coarse grained infection rate.

B. Beyond constant mutation rate

For our multi component model that goes beyond a constant mutation rate, the infection rates β_n evolve according to a biased random walk (see also Methods). Figure S4 shows the infections rate β_n as a function of time. We find a very broad distribution, whose mean increases with time.

We also study the coarse grained infection rate $\beta(t) = \int_0^t \lambda I(t')\mathrm{d}t'$ of our beyond constant mutation rate model. In Fig. S5 we show the infection rate together with the infected for the four different phases that we find in the pandemic. A lethargic (Fig. S5(a)) behavior shows an almost constant mutation rate. The explosive (Fig. S5(b)) and rebound (Fig. S5(c)) phases show a strong sudden increase of the infection rate that coincides with the strong increase in infections. Finally the weak rebound (Fig. S5(d)) has a small constant increase of the infection rate.

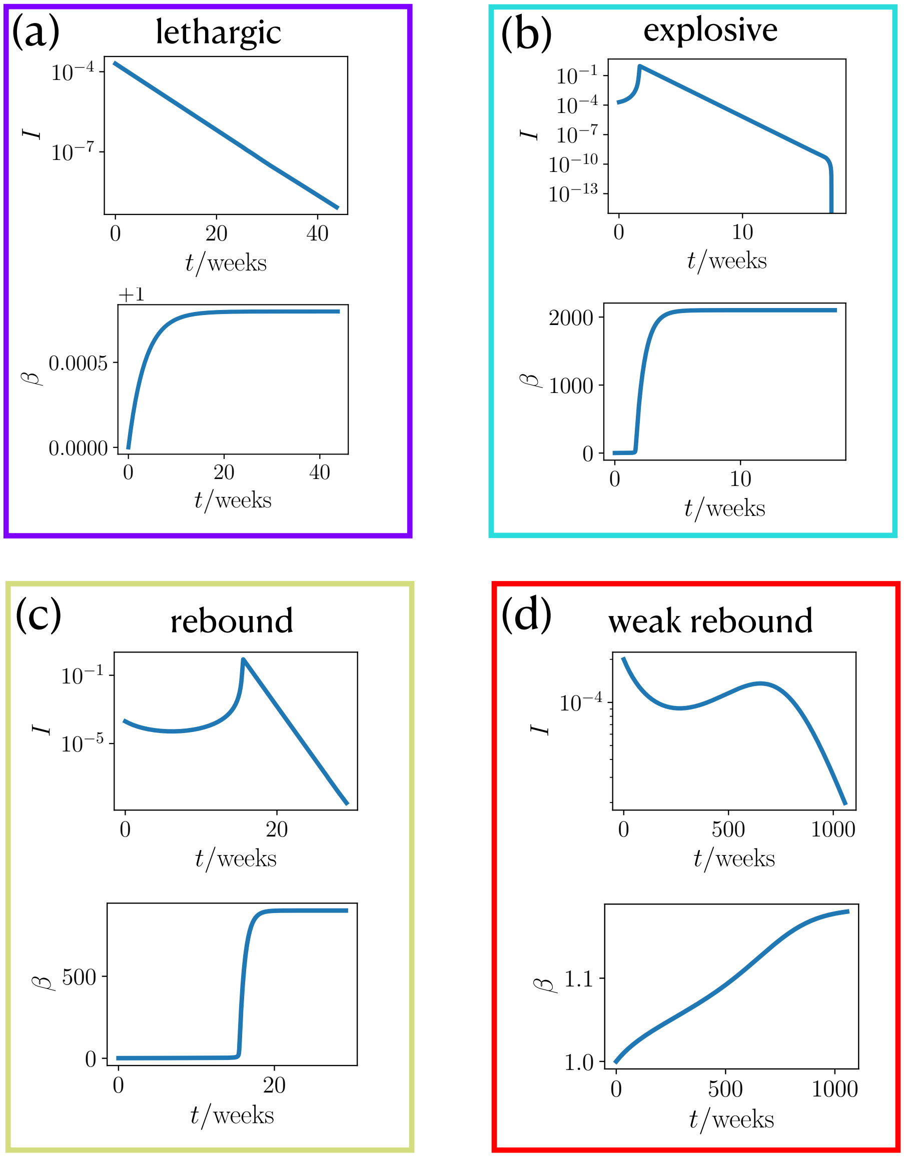

FIG. S5. Coarse grained infection rate (bottom) and infected (top) as a function time for the four different phases: (a)lethargic, (b) explosive, (c) rebound and (d) weak rebound.
